# Supplementary material for: Behavioural and neural signatures of perceptual decision-making are modulated by pupil-linked arousal
Source: eLife. 2019 Mar 18;8:e42541. doi: 10.7554/eLife.42541 (PMC6450670; doi:10.7554/eLife.42541)
Supplement: Supplementary file 1. [file elife-42541-supp1.docx]

|  | Model comparison | | Parameter estimates | | | |
| --- | --- | --- | --- | --- | --- | --- |
|  | *χ^2^* | *p* | *β* | *β SE* | *t* | *p* |
| *BPD* | 5.281849 | 0.021549 | 0.098804 | 0.062248 | 1.587268 | 0.112468 |
| *BPD^2^* | 57.46926 | 3.43E-14 | 0.297062 | 0.048582 | 6.114657 | 9.86E-10 |
| *PR* | 302.0113 | 1.20E-67 | -0.7568 | 0.043115 | -17.5531 | 1.85E-68 |
| *PR^2^* | 118.6822 | 1.23E-27 | -0.48898 | 0.044813 | -10.9114 | 1.22E-27 |
