# Supplementary material for: Behavioural and neural signatures of perceptual decision-making are modulated by pupil-linked arousal
Source: eLife. 2019 Mar 18;8:e42541. doi: 10.7554/eLife.42541 (PMC6450670; doi:10.7554/eLife.42541)
Supplement: Supplementary file 3. [file elife-42541-supp3.docx]

|  | RT | | | | RTcv | | | |
| --- | --- | --- | --- | --- | --- | --- | --- | --- |
| EEG component | β | se | t | p | β | se | t | p |
| Pre-target α Power | 0.19 | 0.07 | 2.87 | 4.5E-03 | -0.03 | 0.06 | -0.42 | 0.68 |
| N2c latency | 7.5E-04 | 0.02 | 0.04 | 0.97 | 0.06 | 0.04 | 1.41 | 0.16 |
| N2c amplitude | 0.04 | 0.03 | 1.25 | 0.21 | 0.04 | 0.06 | 0.73 | 0.47 |
| N2i latency | 0.02 | 0.02 | 1.27 | 0.20 | -0.01 | 0.03 | -0.38 | 0.70 |
| N2i amplitude | 0.04 | 0.03 | 1.27 | 0.20 | 0.01 | 0.06 | 0.25 | 0.81 |
| CPP onset | 0.05 | 0.04 | 1.04 | 0.30 | -0.09 | 0.08 | -1.12 | 0.26 |
| CPP build-up rate | -0.14 | 0.06 | -2.25 | 0.03 | 1.0E-03 | 0.11 | 0.01 | 0.99 |
| CPP amplitude | 0.15 | 0.07 | 2.27 | 0.02 | 0.07 | 0.12 | 0.54 | 0.59 |
| CPP ITPC | -0.18 | 0.04 | -4.43 | 1.3E-05 | -0.26 | 0.06 | -4.13 | 5.4E-05 |
| LHB build-up rate | -0.01 | 0.04 | -0.29 | 0.77 | -0.03 | 0.08 | -0.33 | 0.74 |
| LHB amplitude | 0.05 | 0.06 | 0.86 | 0.39 | 0.03 | 0.09 | 0.27 | 0.78 |
