# Supplementary material for: Behavioural and neural signatures of perceptual decision-making are modulated by pupil-linked arousal
Source: eLife. 2019 Mar 18;8:e42541. doi: 10.7554/eLife.42541 (PMC6450670; doi:10.7554/eLife.42541)
Supplement: Supplementary file 4. — Boldface font indicates parameters that significantly improved the model fit compared to the addition of the neural signal associated with the previous neural processing stage. Red text indicates the parameters that were excluded from the final model during the forward/backward stepwise regression (main text). Final model fits revealed a marginal (conditional) r2 of 4.2% (94.4%) and 11.9% (44.5%) for RT and RTcv, respectively. [file elife-42541-supp4.docx]

|  | RT | | | | RTcv | | | |
| --- | --- | --- | --- | --- | --- | --- | --- | --- |
|  | Model comparison | | Stepwise model selection | | Model comparison | | Stepwise model selection | |
| EEG component | χ^2^ | p | F | p | χ^2^ | p | F | p |
| Pre-target α Power | 0.56 | 0.46 |  |  | 0.01 | 0.90 |  |  |
| N2c latency | 0.83 | 0.36 |  |  | 0.06 | 0.80 |  |  |
| N2c amplitude | **6.18** | **0.013** | **6.38** | **0.012** | 0.87 | 0.35 |  |  |
| N2i latency | 0.10 | 0.75 |  |  | 0.01 | 0.93 |  |  |
| N2i amplitude | 0.33 | 0.57 |  |  | 0.03 | 0.87 |  |  |
| CPP onset | **5.86** | **0.016** | 0.01 | 0.92 | 2.50 | 0.11 |  |  |
| CPP build-up rate | **4.06** | **0.044** | 2.02 | 0.16 | 0.47 | 0.49 |  |  |
| CPP amplitude | 0.01 | 0.91 |  |  | 0.77 | 0.39 |  |  |
| CPP ITPC | **21.49** | **< 0.001** | **28.43** | **< 0.001** | **24.53** | **< 0.001** | **30.37** | **< 0.001** |
| LHB build-up rate | 0.01 | 0.91 |  |  | 2.50 | 0.11 |  |  |
| LHB amplitude | 0.07 | 0.80 |  |  | 0.27 | 0.60 |  |  |
