# Supplementary material for: Behavioural and neural signatures of perceptual decision-making are modulated by pupil-linked arousal
Source: eLife. 2019 Mar 18;8:e42541. doi: 10.7554/eLife.42541 (PMC6450670; doi:10.7554/eLife.42541)
Supplement: Supplementary file 5. [file elife-42541-supp5.docx]

|  | RT | | | | RTcv | | | |
| --- | --- | --- | --- | --- | --- | --- | --- | --- |
| EEG component | β | se | t | p | β | se | t | p |
| Pre-target α Power | 0.07 | 0.07 | 1.00 | 0.32 | -0.06 | 0.08 | -0.75 | 0.46 |
| N2c latency | 0.03 | 0.02 | 1.31 | 0.19 | -0.01 | 0.04 | -0.16 | 0.87 |
| N2c amplitude | 0.06 | 0.03 | 2.17 | 0.03 | 0.04 | 0.06 | 0.71 | 0.48 |
| N2i latency | -2.2E-03 | 0.01 | -0.16 | 0.87 | 0.00 | 0.03 | 0.14 | 0.89 |
| N2i amplitude | 0.02 | 0.03 | 0.46 | 0.65 | -0.05 | 0.07 | -0.64 | 0.52 |
| CPP onset | 0.01 | 0.04 | 0.22 | 0.83 | -0.09 | 0.09 | -0.97 | 0.33 |
| CPP build-up rate | -0.12 | 0.06 | -1.97 | 0.05 | -0.04 | 0.13 | -0.29 | 0.78 |
| CPP amplitude | 0.10 | 0.07 | 1.50 | 0.13 | 0.15 | 0.14 | 1.05 | 0.29 |
| CPP ITPC | -0.19 | 0.04 | -4.80 | 2.6E-06 | -0.39 | 0.07 | -5.40 | 1.71E-07 |
| LHB build-up rate | -0.01 | 0.05 | -0.18 | 0.86 | -0.15 | 0.09 | -1.63 | 0.10 |
| LHB amplitude | 0.01 | 0.05 | 0.26 | 0.79 | 0.05 | 0.09 | 0.53 | 0.60 |
